# Gallic Acid Inhibits the Proliferation and Migration of Ovarian Cancer Cells via Inhibition of the PI3K-AKT Pathway and Promoting M1-Like Macrophage Polarization

**DOI:** 10.1155/ancp/3880719

**Published:** 2025-04-16

**Authors:** Ran Meng, Zhengmao Zhang

**Affiliations:** Department of Gynecology, The Fourth Hospital of Hebei Medical University, No. 12 Jiankang Road, Shijiazhuang 050011, China

**Keywords:** gallic acid, macrophage polarization, migration, ovarian cancer, PI3k-AKT pathway

## Abstract

Ovarian cancer is one of the leading malignant women tumors that causes higher mortality, and immunotherapy has shown high potential in the treatment of advanced ovarian cancer patients by activating and mobilizing the human immune system, which can improve patient prognosis and survival. Natural compounds are a big resource for screening and finding effective lead compounds to treat diseases. Gallic acid (GA) is a natural organic acid with broad-spectrum antibacterial, antiviral, and antitumor effects. In the current study, we aim to explore the effect of GA on ovarian cancer and its underlying mechanisms. The CCK-8 assay was employed to study its anti-proliferation effect and wound healing, and transwell assay was utilized to test the GA effect on cell migration and invasion. The xenograft tumor model was used to evaluate the GA anticancer effect in vivo. The results demonstrated that GA significantly suppresses the proliferation of ovarian cancer cells both in vitro and in vivo, reduces their migration and invasion capability, and enhances macrophage cytotoxicity in the murine ID8 xenograft tumor microenvironment (TME). The mechanism study demonstrated that its anticancer effect and enhancing immunity is stem from inhibiting the PI3k-AKT pathway. In conclusion, GA plays an anticancer effect via blockage of the PI3K-AKT pathway.

## 1. Introduction

Ovarian cancer is one of the worst prognosis malignant tumors among gynecological malignancies. Due to the low early diagnosis rate and prone to recurrence after treatment, clinical workers were plagued to break through the treatment bottleneck of postoperative recurrence in most ovarian cancer patients, especially in patients at advanced stages with metastasis [1]. An immunosuppressive microenvironment forms in specific areas of the peritoneum through the interaction of tumor, immunosuppressive, and stromal cells before ovarian cancer metastasis [2]. The involved changes include tumor cell characteristics, changing abnormal recruitment and function of immune cells, and abnormal distribution and regulatory ability of stromal cells. The metastasis of ovarian cancer is strongly associated with the formation of local immunosuppression in the premetastatic niche [3] and targeted regulation of premetastatic niche-related cells has the potential to improve the treatment response in patients with ovarian cancer.

Macrophages are immune cells inherent in the humoral immunity of the human immune system. Macrophages play an important role in autoimmune, inflammatory responses, and tumor immunity. They participate in physiological processes such as maintaining tissue homeostasis, bacterial defense, and controlling immune responses [4]. Macrophages have high plasticity and heterogeneity. Most macrophages can be categorized into two phenotypes based on their activation status. Classically activated macrophages (M1 type) produce a range of pro-inflammatory and immunostimulatory molecules, including interleukin-1*β* (IL-1*β*) and tumor necrosis factor-*α* (TNF-*α*). They exhibit potent antitumor activity. In contrast, alternatively activated macrophages (M2 type) display a tumor-promoting phenotype and express various anti-inflammatory effector mediators, including IL-10 and TNF-*β* [5, 6]. This helps to form a tumor microenvironment (TME) with more immunosuppressive effects. Consequently, promoting changes in macrophage subpopulations towards the M1 type may become a therapeutic target for ovarian cancer immunotherapy. The CD47/SIRP*α* pathway is a critical mechanism by which tumors evade macrophage detection, and CD47 is a transmembrane protein that is often expressed on the surface of tumor cells. Targeting CD47 in tumor cells has been a promising cancer treatment strategy, and its expression is regulated by several pathways, including the PI3K-AKT pathway [7, 8].

Gallic acid (GA) is an organic acid with broad-spectrum antibacterial, antiviral, and anticancer properties. It is the main component of many traditional Chinese medicines (such as gallnut, GA, dogwood, pomegranate, rhubarb, and so on.) [9]. In addition, GA can boost the immunity [10, 11]. The above evidence suggests that it may be a potential natural compound that can play an important auxiliary treatment. In this study, we explored the effect of GA against ovarian cancer cells in vitro and in vivo, and further explored its related mechanism.

## 2. Methods

### 2.1. Cell Lines

The human ovarian cancer cell lines SKOV3, A2780, and human macrophage cell THP-1, were obtained from the Institute of Basic Medical Sciences Chinese Academy of Medical Sciences. SKOV3 cells were maintained in McCoy's 5A medium (#C3020-0500, VivaCell, Shanghai, CHN) enriched with 10% fetal bovine serum (FBS) and 1% penicillin/streptomycin. The A2780 cells were grown in DMEM medium (#C3020-0500, SIGMA, GER) enriched with 10% FBS and 1% penicillin/streptomycin. The THP-1 cells were grown in RPMI 1640 medium (Hyclone Corporation, USA) supplemented with 10% FBS and a mixture of penicillin and streptomycin.

To evaluate the half-maximal inhibitory concentration (IC_50_) of GA on ovarian cancer cells, GA (#S4603, Selleck) was dissolved in DMSO, diluted, and added to the cell culture. A serial concentration of 0, 2.5, 5, 10, 20, and 40 μM were adopted in the CCK-8 assay.

### 2.2. CCK-8 Assay

A total of 100 μl cell suspension was loaded in a 96-well plate. The loaded concentration was 5 × 10^3^ cells/well. Briefly, cells were suspended in 100 μl medium and inoculated at the logarithmic growth phase. The cells were cultured for 24 h and GA with different concentrations or vehicle control (DMSO) was added to wells. The cells were cultured for another 48 h, and cell activity was measured using a microplate reader (iMark Microplate Absorbance Reader, Bio-Rad, USA). Ten microliters of CCK-8 solution was added to each well, followed by cultured for another 4 h before measurement. The optical density (OD) value at 450 nm was recorded.

### 2.3. Wound Healing Measurement

The measurement was performed on a 6-well plate. 5 × 10^5^ cells were loaded on the culture medium with a horizontal line drawn on the back of the plate in advance. Cells were spread evenly on the culture medium and cultured for 4 h. A sterile pipette tip was used to create a scratch wound in the cell monolayer. The debris on the plate was washed twice with PBS. The migration of cells was observed under an optical microscope 24 h after inoculation. The width of the scratch was measured using ImageJ software (NIH, USA) and the migration rate was calculated as the relative width at 0 h.

### 2.4. Transwell Measurement

The diluted Matrigel gel was added to the upper transwell chamber (#3428, Corning Life Science). A total of 200 μL cells (5 × 10^5^ cells) without serum supplementary were added in the upper chamber and 1 mL medium supplemented with 10% FBS was loaded into the lower compartment of the chamber. The chamber was cultured in an incubator for 48 h, and the noninvasive cells in the upper chamber was removed. The invaded cells were fixed with 4% formaldehyde for 25 min and stained with 0.2% crystal violet for 10 min. After air drying, five fields of view were selected under a high-power microscope (CH30 Microscope, Olympus, JPN) to count invaded cell numbers.

### 2.5. Effect of GA on ID8 Ovarian Cancer in Mouse and M1/M2 Polarization of Macrophages

The murine ID8 cell line was purchased from the Chinese Academy of Medical Sciences Cell Bank (Beijing, China). Female C57BL/6J mice, aged 6 weeks and weighing 18–22 g, were acclimatized for 1 week prior to the experiment. Throughout the study, the mice were housed under controlled environmental conditions of temperature (20 ± 2°C) and a 12-h light/12-h dark cycle. They were fed with a standard pellet diet and provided with water ad libitum [12]. The mice were fasted for 12 h prior to the creation of the tumor model. A 0.2 mL suspension of cancer cell (1 × 10^7^ cells/mL) was subcutaneously injected into the right axilla of each mouse to establish a solid tumor model. Twenty-four hours later, the mice were randomly divided into three groups, with six mice in each group, as follows: two doses of GA (10 and 20 mg/kg/day) groups and an ID8-bearing group (model control group). The mice in the model control group were given an equal volume of double-distilled water daily. All groups underwent continuous treatment for 21 days. Daily observations were made of the animals' living conditions. The tumor volume and body weight changes were monitored. Twenty-four hours after the last administration, all the mice were sacrificed using cervical translocation. The tumor was immediately dissected and weighed, and following the frozen tissue sections were prepared, and the expression patterns of macrophage type (M1) CD86 and M2 surface molecule CD206 were analyzed using immunofluorescence.

### 2.6. Immunofluorescence

The immunofluorescence was used to examine the CD86 and CD206 expression in ID8 tumor tissues from C57BL/6J mice which was described by a previous publication [13]. Briefly, the tumor frozen tissue sections (2 μm thickness) were treated with primary CD86 and CD206 antibodies and incubated (1:100, abcam) overnight, then labeled by Texas Red-Alexa Fluor secondary Antibodies (Thermo Fisher, CA, USA) for 2 h. Later, the panoramic images were captured by a charge-coupled device camera (S610; Hamamatsu Photonics, Tokyo, Japan) connected to the image analysis software (NDP Viewer 2; Hamamatsu Photonics, Tokyo, Japan).

### 2.7. Immunohistochemistry

The protein level of VEGF in tumor tissue was analyzed. The above tumor was fixed in 4% paraformaldehyde and prepared into 6 μm slices after embedding them in wax. Slices were deparaffinized with xylene and alcohol and then hydrated with PBS. Antigen retrieval buffer (C1034, Solarbio) was added and antigens were retrieved by high-pressure method. After adding endogenous peroxidase and washing the slices with PBS, the diluted VEGF antibody (MA5-32038, 1:200, Invitrogen) was added to the slices and incubated at 37°C for 2 h. A sheep anti-rabbit IgG antibody was added and incubated at 37°C for 30 min. The slices were stained with diaminobenzene (DAB) and examined at 200x magnification using a light microscope. The images were captured for immunohistochemistry analysis. The brown color indicated positive staining. Following the methods described by Wang et al. [14], the *H*-score of VEGF in tissues was calculated and recorded.

### 2.8. Quantitative Real-Time PCR (qRT-PCR)

Total RNA was isolated from ovarian cancer cells using TRIzol reagent (Invitrogen, CA, USA). Reverse transcription and qRT-PCR were performed conducted following the protocols provided with the PrimeScript reverse transcript Reagent Kit (Takara, Japan) and SYBR PrimeScript miRNA RT-PCR Kit (Takara, Japan). *β*-Actin served as the internal control, and all the sequences of all primers used are listed in Table 1.

### 2.9. Coculture of Ovarian Cancer and Macrophage Cells

The THP-1 cells (1 × 10^5^ cells/well) were exposed to 100 ng/mL phorbol-12-myristate-13-acetate (PMA, # 524400, Sigma–Aldrich) for 24 h to induce M0 macrophages in a 6-well plate. After that, the medium was discarded, and cells were rinsed twice with PBS before fresh medium was introduced. The A2780 cells which were pretreated with GA (20 μM) for 24 h, were added to the THP-1 cell culture plate and cocultured for another 24 h, in which the cell number ratio of THP-1 to A2780 was 3:1, and GA concentration was maintained at 20 μM in coculture system. After being cocultured for another 24 h, the suspended THP-1 was collected by centrifuge and the biomarker of M1 CD86 was analyzed by flow cytometry and western blot.

### 2.10. Phagocytosis Analysis

Human THP-1 macrophages were seeded at a density of 5 × 10^4^ per well in a 24-well tissue culture plate, maintained in complete RPMI-1640 medium for 24 h prior to the experiment. A2780 cells were labeled with 2.5 μM carboxyfluorescein succinimidyl ester (CFSE) (Sigma-Aldrich, Germany) following the manufacturer's protocol (Invitrogen). Macrophages were maintained in serum-free medium for 2 h prior to the addition of 2 × 10^5^ CFSE-labeled A2780 cells for coculture, meanwhile, the GA (20 μM) was added into the coculture system. Following a 12-h coculture at 37°C, the cells were collected. Macrophages were labeled with APC-conjugated F4/80 (Novus Biologicals), and flow cytometry analysis were conducted using a FACScalibur (BD Biosciences) was performed. A total of 10,000 cells per sample were analyzed. Unstained controls and single-stained cells were prepared for gating purposes. Phagocytosis was quantified as the percentage of F4/80 + CFSE + cells (Q2) among all CFSE + cells (Q2 + Q3), calculated using the formula:

  $$\begin{array}{c} \text{Phagocytosis}\left(\%\right)=\left[Q2/\left(Q2+Q3\right)\right]\times 100\%. \end{array}$$

### 2.11. Western Blot Assay

The culture medium of the cells was removed, and the cells were subsequently lysed, and cultured on ice for 20 min. Cells were further centrifuged at 12,000 rpm for 20 min at 4°C, and the supernatant was collected. Protein concentration was determined using the Bradford method. A total of 30 μg of denatured protein was loaded onto an SDS–PAGE gel (100 g/L). The separated protein samples were transferred to a PVDF membrane, which was then blocked with 5% skimmed milk powder at room temperature for 2 h. The primary antibody for p-PI3K (#PA5-17387, 1:1000, Invitrogen), PI3K (#MA5-14870, 1:1000, Invitrogen), p-AKT (#5012S, 1:1000, Cell Signaling), AKT (#14702S, 1:1000, Cell Signaling), CD86 (#ET1606-50, 1:1000, HuaBio), CD206 (#MA5-35076, 1:1000, Invitrogen), CD47 (#ab193940, 1:1000, abcam), IFN*γ* (#MM700B, 1:1000, Invitrogen), CD8 (#RM1129, 1:1000, abcam), and GAPDH (#ET1601-4, 1:5000, HuaBio) was loaded and incubated overnight at 4°C. The membrane was rinsed with phosphate-buffered saline containing tween (PBST) and HRP-labeled goat anti-rabbit IgG (1:5000) was added and incubated at room temperature for 2 h. The bands were developed by using ECL chemiluminescence reagents, and the protein expressions were scanned and calculated by ImageJ software.

### 2.12. Statistical Analysis

The experimental results are expressed as mean ± standard deviation (SD), and each group of experiments is repeated in triplicate. The comparison between the two groups is conducted using a two-tailed unpaired student's *t*-test and ANOVA analysis was used for two and multiple group comparisons, respectively. Data were presented and analyzed by GraphPad Prism 9.0 software. A *p*-value of less than 0.05 (*p* < 0.05) indicates that the difference is statistically significant.

## 3. Results

### 3.1. GA Inhibits the Proliferation of Ovarian Cancer Cells in a Dose-Dependent Manner

**Figure 1:**
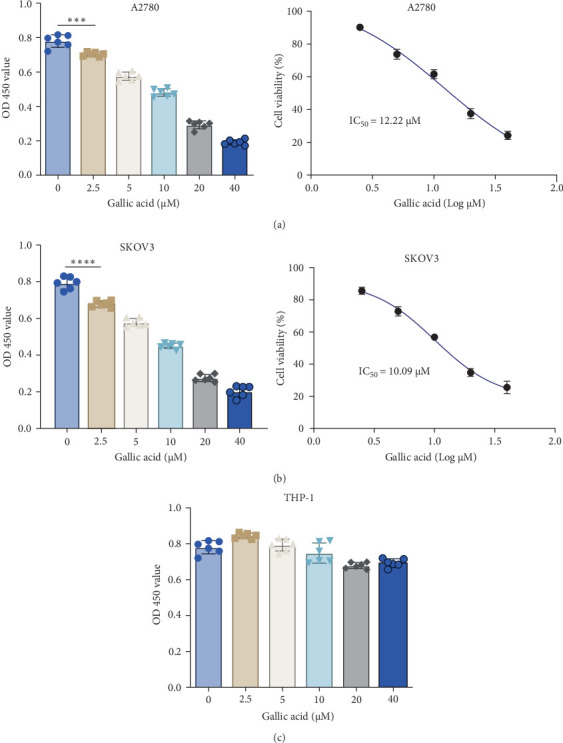
The inhibition of gallic acid (GA) on ovarian cancer A2780 and SKOV3 cells. (A,B) A serial concentration of 0, 2.5, 5, 10, 20, and 40 μM were set, and doses have a good inhibit effect on A2780 and SKOV3 cells. The IC_50_ value of GA on both cells was calculated by GraphPad software, and the calculated values were 12.22 μM and 10.09 μM, respectively. Three dosages of GA, 5, 10, and 20 μM were selected for subsequent experiments. (C) GA was not cytotoxic to human macrophage THP-1 even at 40 μM by CCK-8 assay. *⁣*^*∗∗∗*^*p* < 0.001; *⁣*^*∗∗∗∗*^*p* < 0.0001.

The cytotoxicity of a serial dose of GA against ovarian cancer cells SKOV3 and A2780 was measured, respectively, and results are listed in Figure 1. All current doses have good inhibitory effect on A2780 and SKOV3 cells (Figure 1A,B). The IC_50_ value of GA on both cells was 12.22 μM and 10.09 μM, respectively (Figure 1A,B). Three dosages of GA, 5, 10, and 20 μM were selected for subsequent experiments. Although GA showed cytotoxicity to ovarian cancer cells, the GA was not cytotoxic to human macrophage THP-1 even at 40 μM by CCK-8 assay (Figure 1C).

### 3.2. GA Attenuates the Migratory and Invasive Ability of Ovarian Cancer Cells

**Figure 2:**
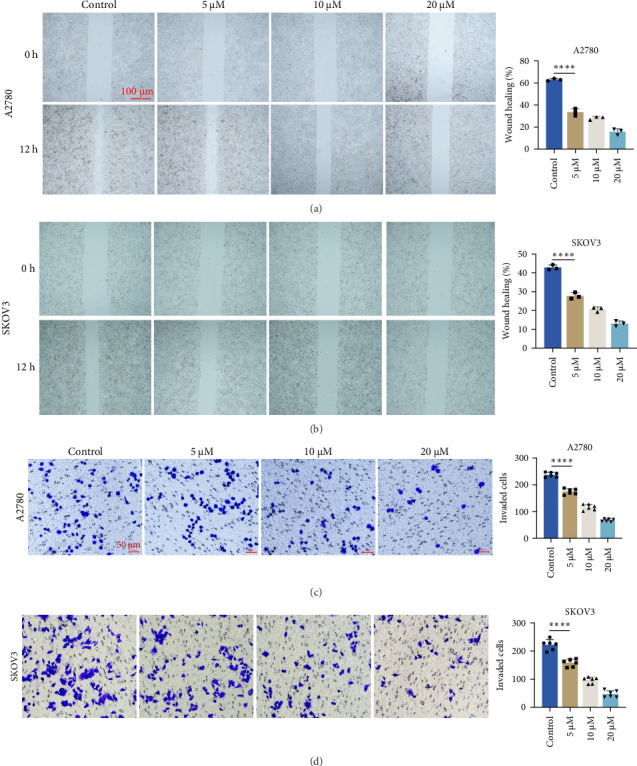
Gallic Acid (GA) inhibits the migration and invasion ability of ovarian cancer cells. (A,B) GA attenuates the migration ability of A2780 and SKOV3 cells. (C,D) The invasion ability of SKOV3 and A2780 cells was inhibited by GA. *⁣*^*∗∗∗∗*^*p* < 0.0001.

The migration capability of ovarian cancer cells was evaluated by wound healing assay, and the invasion ability of ovarian cancer cells was measured by transwell assay. Consistent with the anti-proliferation research results, we observed that GA attenuated the migration (Figure 2A,B) and invasion (Figure 2C,D) of ovarian cancer cells in a dose-dependent manner. As the concentration of GA increases, the migratory and invasive potential of SKOV3 and A2780 cells gradually decreases compared to the control.

### 3.3. The AntiCancer Effect of GA Might be Through the PI3K-AKT Pathway

**Figure 3:**
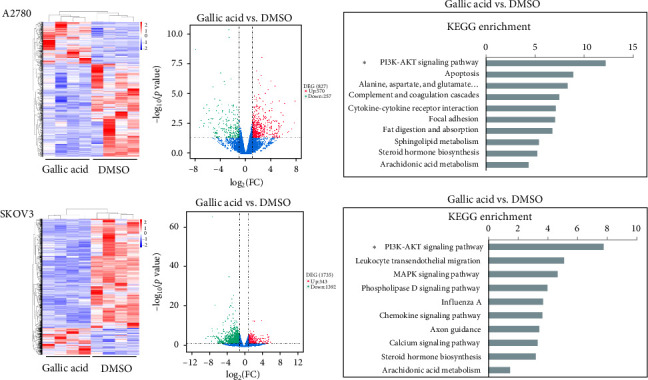
Gallic acid might act via the PI3K-AKT pathway in ovarian cancer. The PI3K-AKT pathway was the most highly enriched downregulated pathway analyzed by KEGG. *⁣*^*∗*^*p* < 0.05.

**Figure 4:**
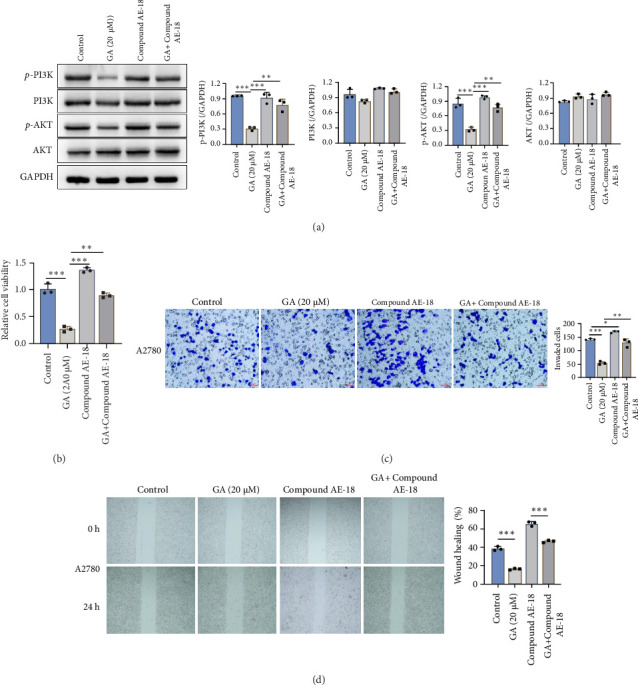
Gallic acid (GA) inhibits ovarian cancer cells via the blockage of PI3K/AKT pathway. (A) GA treatment significantly reduced the protein levels of p-PI3K and p-AKT in A2780 cells, while this inhibition by GA had been reversed by PI3K-AKT activator compound AE-18. (B) AE-18 abolished the GA inhibitory effect on cell proliferation. (C,D). AE-18 abolished the GA inhibitory effect on the invasion (C) and migration (D) of A2780 ovarian cancer cells. *⁣*^*∗∗*^*p* < 0.01; *⁣*^*∗∗∗*^*p* < 0.001.

Aiming to understand the mechanism of the anticancer effect of GA, the cancer cells treated with GA were sent for mRNA sequencing (Novogene, Beijing, China). The KEGG pathway revealed that the PI3K-AKT pathway was the most highly enriched downregulated pathway in both A2780 and SKOV3, which are shown in Figure 3. The PI3K-AKT was widely accepted as a pro-proliferation pathway to enhance cancer cell survival. The activation of the PI3K/AKT signaling pathway is a key that plays a crucial role in leading to higher invasiveness and migration ability of ovarian cancer cells [11, 12], and consistent with mRNA sequencing results, the GA treatment significantly reduced the protein levels of p-PI3K and p-AKT, but not total PI3K and AKT, which are shown in Figure 4A. Aims to further clarify whether GA played its anticancer effect dependent on the PI3K/AKT pathway, the PI3K/Akt/CREB activator 1 (compound AE-18) was added and co-incubated with GA on A2780 cells. As shown in Figure 4A, the inhibition of p-PI3K and p-AKT by GA had been reversed by compound AE-18 confirmed by western blot. The rescue assay also demonstrated that AE-18 abolished the GA's inhibitory effect on cell proliferation (Figure 4B), invasion (Figure 4C), and migration (Figure 4D). These results suggest GA inhibited proliferation, migration, and invasion of ovarian cancer cells dependent on the blockage of the PI3K/AKT pathway.

### 3.4. GA Enhances the Phagocytic Ability and M1 Polarization of Macrophages in Tumor Coculture System via Downregulating CD47 Expression Through the PI3K-AKT Pathway

**Figure 5:**
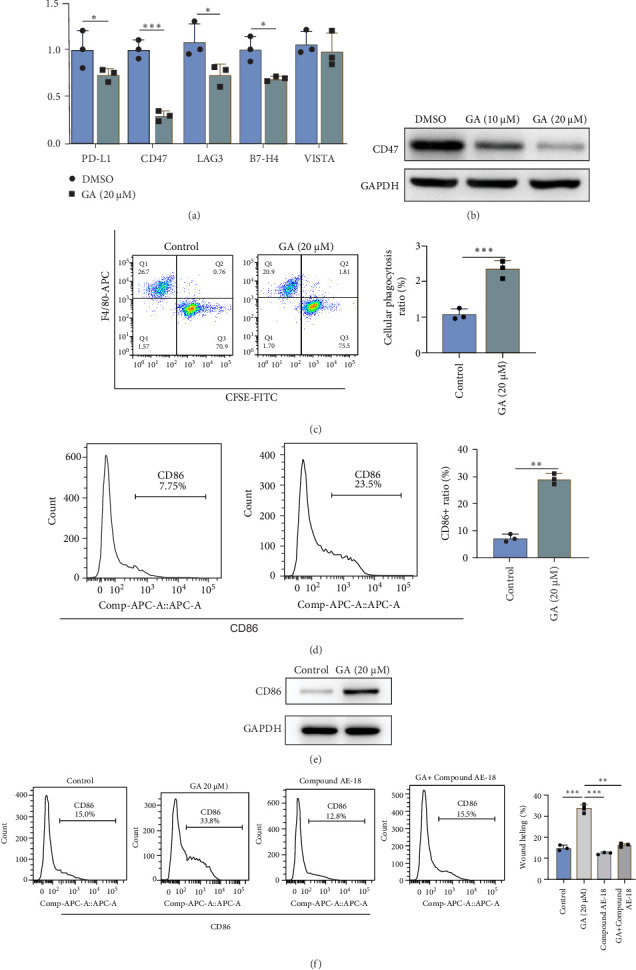
Gallic Acid (GA) enhances the phagocytic ability and M1 polarization of THP-1 in the tumor co-culture system via downregulating CD47 of A2780. (A) CD47 was the most downregulated immune checkpoint gene by GA. (B) GA significantly reduced CD47 protein levels in A2780 cells dose-dependently determined by western blot. (C) GA treatment significantly increased the phagocytic effect of THP-1 in the coculture system by flow cytometry analysis. (D) CD86 (M1 biomarker) positive ratio was significantly upregulated by adding GA in THP-1 and A2780 coculture system. (E) GA increased the CD86 protein in the level of THP-1 by western blot analysis. (F) AE-18 significantly abolishes increased CD86+ macrophage percentage induced by GA by flow cytometry analysis. *⁣*^*∗*^*p* < 0.05; *⁣*^*∗∗*^*p* < 0.01; *⁣*^*∗∗∗*^*p* < 0.001.

Deng et al. [11] reported GA could block several immune checkpoint proteins' efficacy recently, which suggests that the anticancer activity of GA is also, to some extent, related to enhancing immunity. By searching several important immune checkpoint gene expressions using mRNA sequencing data from A2780, we found that the mRNA level of CD47 was most downregulated by GA (Figure 5A), and western blot also confirmed that GA significantly reduced CD47 protein levels in A2780 cells dose-dependently (Figure 5B). Aims to confirm whether the decreasing CD47 caused by GA could enhance the phagocytic ability of macrophages to cancer cells, the A2780 cells and THP-1 were co-cultured to examine the effect of GA (20 μM) on cell phagocytic ability. Flow cytometry revealed that GA treatment markedly enhanced the phagocytic effect of THP-1 in the coculture system (Figure 5C). Furthermore, in the coculture system, flow cytometry demonstrated that CD86 (M1 biomarker) positive ratio was significantly upregulated by adding GA in the coculture system, and western blot also obtained consistent results (Figure 5D,E). The enhancing polarization to M1 induced by the downregulation of CD47 was also reported to be regulated by the PI3K-AKT pathway [15], therefore, the rescue assay was performed to determine whether the M1 polarization caused by GA in A2780 was also dependent on PI3K-AKT pathway using the compound AE-18. As shown in Figure 5F, in the THP-1 and A2780 coculture system, the addition of compound AE-18 significantly abolishes increased CD86+ macrophage percentage induced by GA treatment.

### 3.5. GA Inhibits the ID8 Tumor Growth In Vivo Through the PI3K-AKT Pathway and Regulates the TME

**Figure 6:**
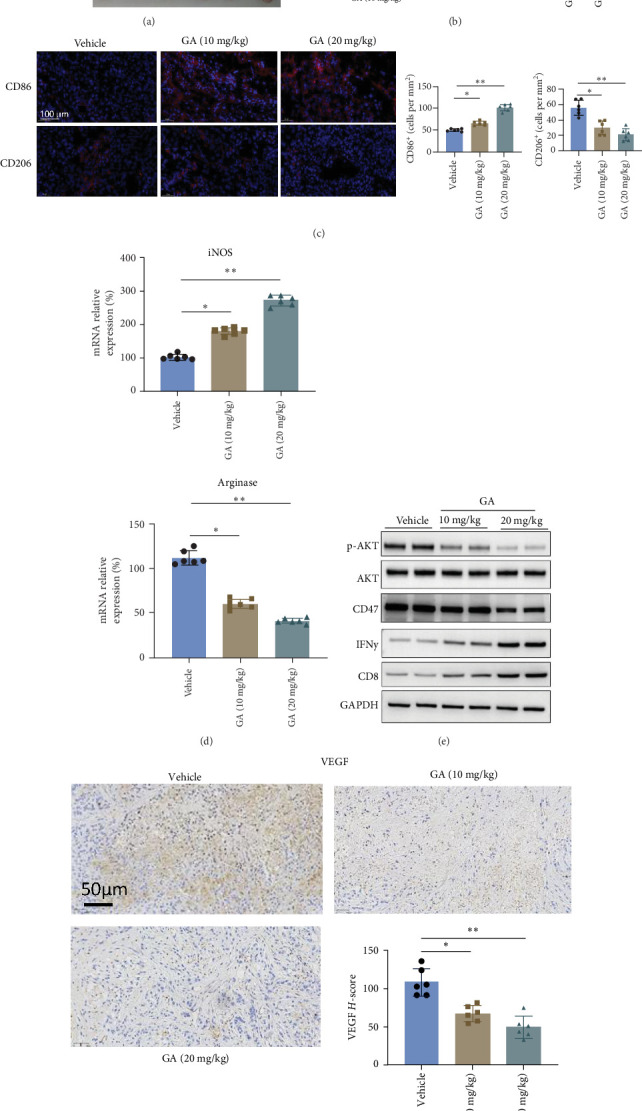
Gallic acid (GA) inhibits the tumor growth of murine ovarian cancer ID8 cells in mice. (A,B) GA reduced tumor volume and weight in C57BL/6J tumor-bearing mice. (C) GA treatment remarkably reduced the M2 marker CD206 positive cells infiltration into the tumor tissues, while infiltrated CD86+ cells as M1 biomarker increased instead in the tumor microenvironment. (D) GA reduced the mRNA levels of the M2 key biomarker Arginase and increased the mRNA expression of the M1 biomarker iNOS. (E) GA inhibits PI3K-AKT-CD47 axis-related proteins in tumor tissue. The protein levels of p-AKT and CD47 were significantly reduced while the IFN*γ* and CD8 were significantly upregulated by GA. (F) GA reduced the protein level of pro-angiogenesis VEGF in tumor tissues dose-dependently. *⁣*^*∗*^*p* < 0.05; *⁣*^*∗∗*^*p* < 0.01; *⁣*^*∗∗∗*^*p* < 0.001.

Murine ovarian cancer ID8 cells were injected subcutaneously into the right axilla of female C57BL/6J mice to explore whether GA could inhibit ovarian cancer growth in vivo. The tumor-bearing mice following GA treatment exhibited reduced tumor volume and weight in a dose-dependent manner (Figure 6A,B). Moreover, by immunofluorescence, we demonstrated that GA treatment remarkably reduced the M2 CD206+ cell number in the tumor tissues, while the M1 CD86+ cell number increased in the TME (Figure 6C). Consistent with immunofluorescence, qRT-PCR further confirmed that GA treatment significantly reduced the mRNA levels of the M2 key biomarker Arginase, and increased the mRNA expression of another M1 biomarker iNOS (Figure 6D).

Aiming to confirm that suppression of the PI3K-AKT-CD47 pathway also contributes to the GA anticancer effect in vivo, whole protein was isolated from tumor tissues, and a western blot was performed. As shown in Figure 6E, the protein levels of p-AKT and CD47 were significantly reduced in tumor tissues dose-dependently. The effects of macrophage polarization on the TME are complex. M2 macrophages generally could promote energy balance and angiogenesis within tumors, while the tumor-suppressive functions of M1 macrophages involve the activation and recruitment of natural killer cells and cytotoxic lymphocytes CD8+ cells [16, 17]. Therefore, we examined these possible changes caused by increased M1 polarization. As shown in Figure 6E, the protein levels of IFN*γ* and CD8 in tumor tissues were remarkably increased confirmed by western blot, which partially reflected the activation of cytotoxic CD8+ T cells. Besides that, a significantly reduced expression of VEGF in tumor tissue, an angiogenesis marker, was noticed by GA treatment dose-dependently (Figure 6F).

## 4. Discussion

Ovarian cancer is one of the most prevalent malignant tumors among women. In recent years, its incidence rate has increased significantly and has been predicted to increase continuously in China [18]. A low early diagnosis rate and susceptibility to recurrence after treatment are the two most important influencing factors for poor prognosis in ovarian cancer patients. Tumors are a complex series of organisms that originate from normal cells. The immune response of tumors is an important process in the occurrence and development of tumors. After detachment from the monitoring of the body's immune system, the aggressive biological behavior of tumors will further accelerate, thereby driving tumor proliferation, invasion, and metastasis [19]. Consequently, understanding the mechanism of immune escape plays an important role in tumor treatment, especially immunotherapy. The mechanism of tumor immune escape is mainly related to the modifications of tumor cells themselves and changes in the tumor immune microenvironment. The TME comprises both cellular and noncellular components, including adaptive immune cells such as CD8+T, CD4+T, Tregs, and B cells. Most immune cells have dual antitumor and tumor-promoting effects [20]. Macrophages have functional plasticity and can repeatedly change their functional characteristics according to environmental changes [21]. This feature also enables them to play a role in tumor treatment. During the development of tumors, M1 macrophages secrete cytokines such as TNF-*α*, IL-6, andIL-12 to exert antigen presentation and regulate the body's immune response to exert antitumor immune effects.

The antitumor effect and immunomodulatory potential of GA have attracted widespread attention. Currently, research mainly focuses on its direct effects on ovarian cancer cells, including inhibiting proliferation and invasion, inducing cell apoptosis, and enhancing sensitivity to chemotherapy drugs. In this study, we evaluated its effect on the immune microenvironment regulation of ovarian cancer, mainly exploring whether GA can further inhibit the malignant behavior of ovarian cancer by regulating the polarization of macrophages. As expected, we found that GA could promote M1-like macrophage polarization in ovarian cancer cells by reducing the CD47 expression in ovarian cancer cells. Our analysis revealed that the inhibitory effect of ovarian cancer relies on, or at least partially on immune regulation. M1-type macrophages typically release immune-stimulating cytokines, chemokines, and effector molecules, such as IL-12, IL-1, and TNF-*α*, which are mainly involved in the Thl-type immune response. M2-type macrophages exhibit a broad spectrum of anti-inflammatory and immunosuppressive factors, such as IL-10, TGF-*β*, CCL23, and CXCR2, which mainly participates in the Th2-type immune response [22]. Deng et al. [11] reported that 31 small molecule compounds, including GA, can downregulate the expression of Treg's key transcription factor FOXP3, thereby, altering the tumor's immunosuppressive environment and slowing down the progression of colorectal cancer. Combined with our research findings, these results provide new strategies and ideas for immunotherapy that potentially target tumor-infiltrating Treg cells for clinical treatment.

**Figure 7:**
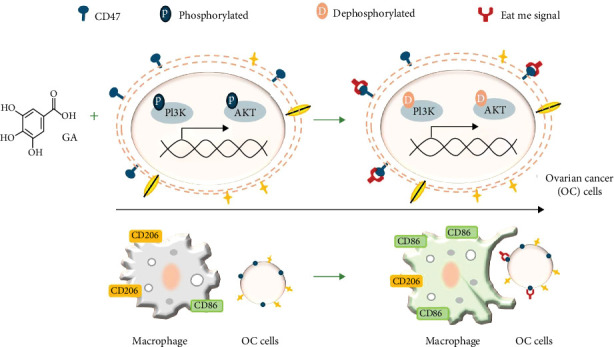
The underlying mechanism of the anticancer effect of gallic acid against ovarian cancer.

The phosphatidylinositol 3 kinase/protein kinase *B* (PI3K/AKT) pathway is closely related to the mitogen-activated protein kinase (MAPK) pathway and can participate in many basic cellular processes, including the regulation of cell survival, growth, proliferation, blood vessel formation, gene expression, translation, and metabolic processes [23]. It is also an important and widely studied intracellular signaling pathway in tumor progression. Research has found that excessive activation of the PI3K/AKT pathway is closely related to the proliferation, invasion, metastasis, and drug resistance of epithelial ovarian cancer [23, 24]. Moreover, the Akt pathway congregates inflammatory and metabolic signals and subsequently regulates macrophage responses modulating their activation phenotype [25]. The metastatic characteristic of ovarian cancer is that seemingly limited tumors spread to the peritoneum, greater omentum, and retroperitoneal lymph nodes. PI3K-Akt signaling plays multiple roles in controlling epithelial–mesenchymal transition (EMT), including cross-talk with extracellular signal-regulated kinase (ERK), c-Jun N-terminal kinase (JNK), and an effective activator of p38 MAPK [26, 27]. These pathways are involved in the invasion and metastasis of ovarian cancer to varying degrees. Consistent with other reported results [28, 29], our study also further confirmed that GA significantly inhibits the PI3K/Akt signaling pathway. Ashrafizadeh et al. reported that GA suppresses the expression of molecular pathways associated with cancer development including PI3K/Akt [30], and in the current study, we further proved that GA's enhancing M1 polarization depends on PI3K-AKT-CD47 axis, and the possible underlying mechanism has been summarized in Figure 7.

## 5. Conclusion

Our analysis reveals that GA could inhibit the proliferation, migration, and invasion ability of ovarian cancer cells, as well as its M1 polarization-promoting effect on macrophages within the TME via the blockade of PI3K-AKT pathway.

## Figures and Tables

**Table 1:** qRT-PCR primer sequence.

Gene	Sequence
Arg1	F: 5′- TCATCTGGGTGGATGCTCACAC-3′
	R: 5′- GAGAATCCTGGCACATCGGGAA-3′
iNOS	F: 5′- CAGCGGGATGACTTTCCAA -3′
	R: 5′- AGGCAAGATTTGGACCTGCA -3′
*β*-Actin	F: 5′- CACCATTGGCAATGAGCGGTTC -3′
	R: 5′- AGGTCTTTGCGGATGTCCACGT-3′

## Data Availability

The datasets are available from the corresponding author upon reasonable request.
